# Polymorphism of the parasite lactate dehydrogenase gene from *Plasmodium vivax* Korean isolates

**DOI:** 10.1186/1475-2875-12-166

**Published:** 2013-05-21

**Authors:** Hyun-Il Shin, Jung-Yeon Kim, Won-Ja Lee, Youngjoo Sohn, Sang-Wook Lee, Yoon-Joong Kang, Hyeong-Woo Lee

**Affiliations:** 1 Division of Malaria and Parasitic Diseases, National Institute of Health, Korea Centers for Disease Control and Prevention, Osong 363-951, Republic of Korea; 2 Department of Anatomy, College of Korean Medicine, Institute of Korean Medicine, Kyung Hee University, Seoul 130-701, Republic of Korea; 3 Department of Pathology, College of Medicine, University of Florida, J-566, 1275 Center Drive, Gainesville, USA; 4 Department of Biomedical Science, Jungwon University, Goesan Chungbuk 367-805, Republic of Korea

## Abstract

**Background:**

Assaying for the parasitic lactate dehydrogenase (pLDH) is widely used as a rapid diagnostic test (RDT), but the efficacy of its serological effectiveness in diagnosis, that is antibody detection ability, is not known. The genetic variation of Korean isolates was analysed, and recombinant protein pLDH was evaluated as a serodiagnostic antigen for the detection of *Plasmodium vivax* malaria.

**Methods:**

Genomic DNA was purified, and the pLDH gene of *P. vivax* was amplified from blood samples from 20 patients. The samples came from five epidemic areas: Bucheon-si, Gimpo-si, and Paju-si of Gyeonggi Province, Gangwha-gun of Incheon metropolitan city, and Cheorwon-gun of Gangwon Province, South Korea, from 2010 to 2011. The antigenicity of the recombinant protein pLDH was tested by western blot and enzyme-linked immunosorbent assay (ELISA).

**Results:**

Sequence analysis of 20 Korean isolates of *P. vivax* showed that the open reading frame (ORF) of 951 nucleotides encoded a deduced protein of 316 amino acids (aa). This ORF showed 100% identity with the *P. vivax* Belem strain (DQ060151) and *P. vivax* Hainan strain (FJ527750), 89.6% homology with *Plasmodium falciparum* FCC1_HN (DQ825436), 90.2% homology with *Plasmodium berghei* (AY437808), 96.8% homology with *Plasmodium knowlesi* (JF958130), and 90.2% homology with *Plasmodium reichenowi* (AB122147). A single-nucleotide polymorphism (SNP) at nucleotide 456 (T to C) was also observed in the isolate from Bucheon, but it did not change in the amino acid sequence. The expressed recombinant protein had a molecular weight of approximately 32 kDa, as analysed by sodium dodecyl sulphate-polyacrylamide gel electrophoresis (SDS-PAGE) analysis. Of the 40 *P. vivax* patients, 34 (85.0%) were positive by ELISA.

**Conclusions:**

The pLDH genes of 19 isolates of *P. vivax* were identical, except one for SNP at nucleotide 456. This observation indicates that this gene is relatively stable. Based on these results, the relationship between antibody production against pLDH and the pattern of disease onset should be investigated further before using pLDH for serodiagnosis.

## Background

Global figures for deaths caused by malaria range from 1.5 to 2.7 million each year, most of which are children under five years of age and pregnant women. Most of the deaths are caused by *Plasmodium falciparum*[1,2]. The clinical diagnosis of malaria still relies upon the identification of malaria parasites in Giemsa-stained blood smears of peripheral blood. Therefore, microscopic observation of the *Plasmodium* species is regarded as the “gold standard” for malaria diagnosis. Despite the simplicity and low cost, such a diagnostic technique is not always available [3]. Rapid diagnostic tests (RDTs) have been introduced to overcome time constraints, a lack of trained personnel in remote or isolated areas, and the low sensitivity when diagnosing malaria infections with a low level of parasitaemia [4]. These lateral-flow immunochromatographic tests detect specific antigens that are produced by malaria parasites and are rapid and simple to carry out without electricity, specific equipment or intensive training [5-8]. To detect *Plasmodium*, monoclonal antibodies against parasite lactate dehydrogenase (pLDH), histidine-rich protein-2 (HRP-2), and aldolase are widely used. The genetic diversity of HRP-2 is known to partly influence the sensitivity of RDT. pLDH catalyzes the inter-conversion of lactate into pyruvate. Therefore, this enzyme is essential for energy production in *Plasmodium* species. The level of pLDH in the blood has been directly linked to the level of parasitaemia [9-12]. pLDH (L-lactate: NAD + −oxidoreductase, EC 1.1.1.27) is the one of the first malaria parasite enzymes that was shown to be electrophoretically and kinetically distinct from a human enzyme [13,14]. Glucose utilization in *P. falciparum*-infected erythrocytes is as much as 100 times the rate observed in uninfected erythrocytes [15]. pLDH plays an important role in regulating glycolysis and in balancing the reduced/oxidized state of the malaria parasites [16,17]. Using RDTs of monoclonal antibodies against the pLDH antigen, sensitivity was over 90%, with high parasite density of *P. falciparum*. However, the sensitivity decreased to under 70% in parasitaemia of less than 50 parasites/μl [6,18,19].

The creation of diagnostic tools and methods for asymptomatic and low parasitaemia patients has been attempted by the malaria team of the Korean National Institute of Health (KNIH). To accomplish this task, genetic variations of *P. vivax* pLDH were investigated to identify the typical strain of Korean isolates, and its recombinant protein was evaluated as an antibody detection tool whether it could compensate for the missing cases by antigen detection with RDTs which showing low antigen detection ability in low parasite density.

## Methods

### Blood sample collection

Patients with clinically suspected malaria attending the Public Health Centers in Gangwha-gun, Gimpo-si, Bucheon-si, and Paju-si of Gyeonggi Province and Cheorwon-gun of Gangwon Province, South Korea from 2010 to 2011 were examined for malaria parasites. Approximately 3 ml of blood was collected from each symptomatic patient. Thin and thick blood smears were prepared for microscopic examination. Blood samples were transported to the Korean National Institute of Health (KNIH), where sera were separated and stored at −20°C for future analysis. Informed consent was obtained from all patients, and all samples were collected under human use protocols that have been reviewed and approved by the Human Ethics Committee of the National Institute of Health (Osong, Korea).

### Amplification of pLDH

For the purpose of the expression of the pLDH gene, genomic DNA was extracted from the whole blood of a malaria patient using a QIAamp Blood Kit (Qiagen, Hilden, Germany). PCRs were performed using AccuPower PCR Premix (Bioneer, Taejeon, Korea), 50 ng of purified genomic DNA, and 40 pmoles each of forward (pLDH-F1; 5′-GGA TCC GCT ACT CAG AGG GAG GTG CTC GTC GAA ATC-3′) and reverse primers (pLDH-R1; 5′-GCA TGC GAG GCA GTA CTC TCC GCA GTC CGG ATC AGT-3′), and the total volume was adjusted to 20 ml with distilled water. The thermocycler conditions were as follows: denaturation at 94°C for 5 min; 35 cycles of 1 min at 94°C, 1 min at 58°C and 2 min at 72°C; and incubation at 72°C for 5 min. All of the PCR products were analysed on a 1.0% agarose gel, confirmed under a UV transilluminator and purified with a Qiagen plasmid mini kit (Qiagen). The purified PCR products were ligated into a pCR2.1 cloning vector (Invitrogen, Carlsbad, CA, USA) and then transformed into *Escherichia coli* Top10 according to Invitrogen’s procedures.

### DNA sequencing and analysis

**Figure 1:**
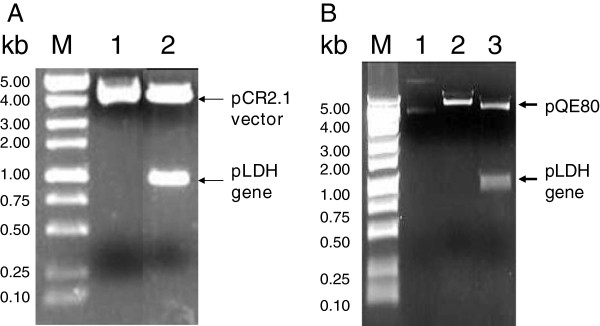
**A) The conformation of cloned pCR-pLDH containing the pLDH gene by digestion with *Eco*RI.** M, Molecular size marker; lane 1, non-inserted clone; lane 2, pLDH gene inserted clone (pVpLDH). **B**) Confirmation of pLDH gene in *Eschelichia coli* DH5α by restriction enzyme digestion with *Eco*RI and *Hin*dIII. M, Molecular size marker; lane 1, Undigested Plasmid; lane 2, *Bam*HI digested Plasmid; lane 3, *Eco*RI and *Hin*dIII digested plasmid.

The PCR product inserted into *E. coli* Top10 was selected for on ampicillin- and 5-bromo-4-chloro-indolyl-β-D-galactopyranoside (X-gal)-containing medium. To confirm transformants, gel electrophoresis was performed after *Eco*RI digestion (Figure 1A) of a plasmid prepared with a Qiagen plasmid mini kit, according to the protocol supplied by the manufacturer. The pLDH gene sequence was determined using an ABI PRISM dye terminator cycle sequencing ready reaction kit FS (Perkin Elmer, Cambridge, MA, USA) according to the supplied manual. M13 reverse and M13 forward (−20) primers were used in sequencing. Nucleotide and deduced amino acid sequences were analysed using EditSeq and Clustal in the MegAlign program, a multiple alignment program in the DNASTAR package (DNASTAR, Madison, WI, USA). The internet-based BLAST search program of the National Center for Biotechnology Information (NCBI) was used to search protein databases. The gene sequences of pLDH from the Korean isolates were deposited in GenBank (Accession No. JX865768-JX865780, JX872274-JX872280).

### Construction of the pLDH expression vector

For the expression of the pLDH gene of the PvKtype19 type strain in *E. coli*, the gene fragment was amplified from the DNA of blood samples that were confirmed to be infected with *P. vivax* as described above and which had *Bam*HI and *Sph*I sites on their 5’ ends. Amplified PCR products were digested with *Bam*HI and *Sph*I, purified with a Qiagen Gel Extraction Kit after running on an agarose gel and integrated into the *Bam*HI and *Sph*I cleavage sites of a pQE80 expression vector (Qiagen). The resulting plasmid was subsequently used for the expression of the pLDH recombinant protein in *E. coli* DH5α. Transformants were confirmed by gel electrophoresis of plasmid DNA after restriction enzyme digestion with *Eco*RI and *Hin*dIII (Figure 1B).

### Expression and purification of recombinant pLDH protein

Expression of the pLDH recombinant protein was induced in *E. coli* with isopropyl-1-thio-β-D-galactopyranoside (IPTG). A total of 1 mM IPTG was added to cultures of *E. coli* DH5α (pVKtype19) grown to the logarithmic phase in liquid Luria Betani (LB) medium containing 100 μg/ml ampicillin to induce expression of the target protein. Purification of pLDH recombinant protein was carried out using immobilized metal ion affinity chromatography [20]. The purification was performed under native conditions according to the manufacturer’s protocol (Qiagen). Proteins were analysed by sodium dodecyl sulphate-polyacrylamide gel electrophoresis (SDS-PAGE) after each purification step.

### Western blot analysis

Recombinant pLDH protein was separated on a 12% SDS-PAGE gel and was then transferred to a nitrocellulose membrane. After the transfer, the membrane was cut into strips and blocked for nonspecific binding with 5% skim milk for 12 hours at 4°C. The membrane was then washed in PBS with 0.15% Tween 20 for 3 × 10 min. The strips were allowed to react with the sera from malaria patients or from uninfected people (diluted 1:100, vol/vol) for four hours and then washed using the procedure described above. The membrane was subsequently incubated with diluted peroxidase-conjugated goat anti-human IgG secondary antibody (1:1,000, v/v) (Sigma) for three hours at room temperature. For colour development, a solution containing 0.2% diaminobenzidine and 0.02% H_2_O_2_/PBS was applied to each well [21].

### Enzyme-linked immunosorbent assay

Sera from patients infected with *P. vivax* were analysed for the presence of antigen-specific antibodies using 96-well plates coated with 0.5 mg/ml purified recombinant protein that had been expressed in *E. coli* and dissolved in phosphate-buffered saline (PBS, pH 7.4) overnight at 4°C. Malaria patient serum was diluted 1:100 (v/v) in blocking buffer (0.25% PBS-Tween 20 with 1% bovine serum albumin, pH 7.4) and incubated for one hour, followed by incubation with peroxidase-conjugated goat anti-human IgG secondary antibody at a 1:1,000 dilution (v/v, Sigma). Optical density was measured with a spectrophotometer at 405 nm (Molecular Devices, Sunnyvale, CA, USA) [22]. Samples were regarded as positive when sera were over the cut-off value, which was calculated as the mean + 2 X the standard deviation (SD) of the negative controls.

## Results

### Sequence variation of pLDH genes from *Plasmodium vivax* Korean isolates

**Figure 2:**
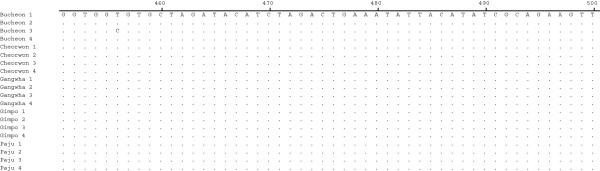
**Comparing the single-nucleotide polymorphism (SNP) of the pLDH gene among 20 *Plasmodium vivax *Korean isolates.** All amino acid sequences were deposited in GenBank (http://WWW.ncbi.nlm.nih.gov/nuccore, Accession No. JX865768-JX865780, JX872274-JX872280).

**Figure 3:**
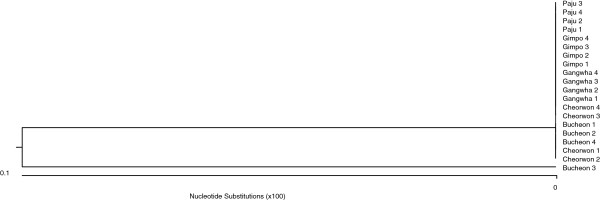
**Nucleotide sequence differences in pLDH genes from *Plasmodium vivax *Korean isolates.** The nucleotide sequences of 20 *P. vivax* Korean isolates were aligned. Computer analysis was performed using the multiple sequences alignment tool of MegAlign. All nucleotide sequences were deposited in GenBank BLAST (http://WWW.ncbi.nlm.nih.gov/nuccore, Accession No. JX865768-JX865780, JX872274-JX872280).

**Figure 4:**
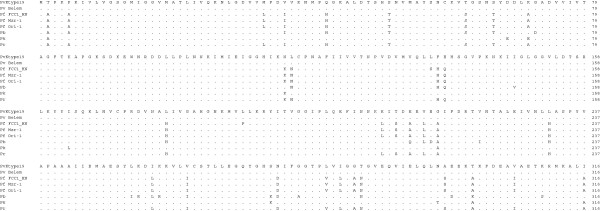
**Multiple amino acid sequence alignment of pLDH among *Plasmodium *species.** The deduced amino acid sequence of the PvKtype19 type strain in *P. vivax* Korean isolates was aligned with those from other *Plasmodium* species. Computer analysis was performed using the multiple sequences alignment of MegAlign. All amino acid sequences were obtained from GenBank BLAST (http://WWW.ncbi.nlm.nih.gov/nuccore). Pv Belem (*P. vivax,* Accession; DQ060151), Pf FCC1_HN (*P. falciparum*, DQ825436), Pf Mzr-1 (*P. falciparum*, JN54719), Pf Ori-1 (*P. falciparum*, JN547218), Pb (*P. berghei*, AY437808), Pk (*P. knowlesi* strain H, JF958130), and Pr (*P. reichenowi*, AB122147).

**Figure 5:**
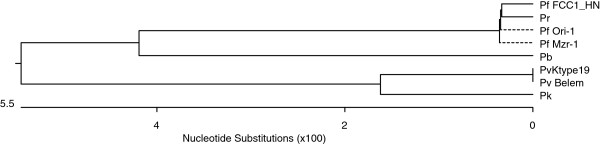
**Amino acid sequence differences in pLDH among *Plasmodium *species.** The deduced amino acid sequence of the PvKtype19 type strain in *P. vivax* Korean isolates was aligned with those from other *Plasmodium* species. Computer analysis was performed using the multiple sequences alignment tool of MegAlign. All amino acid sequences were obtained from GenBank BLAST (http://WWW.ncbi.nlm.nih.gov/nuccore). Pv Belem (*P. vivax,* Accession; DQ060151), Pf FCC1_HN (*P. falciparum*, DQ825436), Pf Mzr-1 (*P. falciparum*, JN54719), Pf Ori-1 (*P. falciparum*, JN547218), Pb (*P. berghei*, AY437808), Pk (*P. knowlesi* strain H, JF958130), and Pr (*P. reichenowi*, AB122147).

**Figure 6:**
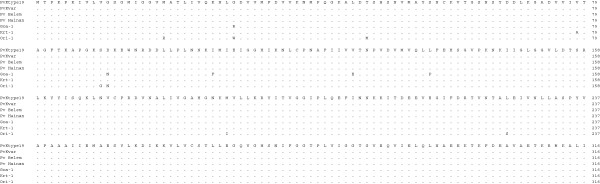
**Multiple amino acid sequence alignment of pLDH among *Plasmodium vivax*.** The deduced amino acid sequence of the PvKtype19 type strain in *P. vivax* Korean isolates was aligned with those from other *Plasmodium* species. Computer analysis was performed using the multiple sequences alignment tool of MegAlign. All amino acid sequences were obtained from GenBank BLAST (http://WWW.ncbi.nlm.nih.gov/nuccore). PvKtype19 (Korean type strain), PvKvar (Variant form of Korean isolate), Pv Belem (*P. vivax,* Accession; DQ060151), and Pv Hainan (*P. vivax*, FJ527750).

**Figure 7:**
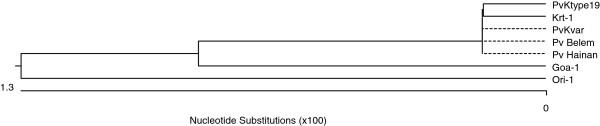
**Phylogenetic relationships among the pLDH of several strains of *Plasmodium vivax*.** Computer analysis was performed using the multiple sequences alignment tool of MegAlign. All amino acid sequences were obtained from GenBank BLAST (http://WWW.ncbi.nlm.nih.gov/nuccore). PvKtype19 (Korean type strain), PvKvar (Variant form of Korean isolate), Pv Belem (*P. vivax,* Accession; DQ060151), Pv Hainan (*P. vivax*, FJ527750), Ori-1 (*P. vivax,* JN547221), Krt-1 (*P. vivax*, JN547225), and Goa-1 (*P. vivax,* JN547223).

The geographical locations of blood sample collection were Gangwha (37.31 N 125.33E) of the Incheon metropolitan city, Gimpo-si (37.33 N 126.48E), Bucheon (37.29 N 126.46E), Paju (37.88 N 126.76E) of Gyeonggi Province, and Cheorwon (38.10 N 127.30E) of Gangwon Province. Four blood samples infected with indigenous *P. vivax* were collected from each city during 2010–2011. The pLDH gene was amplified from the genomic DNA of 20 *P. vivax* Korean isolates. Amplification of the pLDH gene yielded a product of approximately 950 base pairs. After purification, the DNA fragment was ligated into the pCR 2.1 cloning vector (3.9 kb). The plasmid containing the PCR product was named pVpLDH (5.0 kb) and was used for DNA sequence analysis (Figure 1). Based on DNA sequencing, the cloned pLDH gene was 951 bp and consisted of 316 amino acids that were deduced by DNASIS. One single-nucleotide polymorphism (SNP) was detected at base pair 456 (n = 1), from T to C, in the Bucheon 3 isolate (isolated on Sep. 14th 2010) designated as PvKvar (Figures 2 and 3), but it did not change in the amino acid sequence. Therefore, of the 20 Korean isolates of pLDH, 19 isolates had the same DNA and amino acid sequences as *P. vivax* Belem (DQ060151). These isolates were designated as type strain PvKtype19. When the amino acid sequence of PvKtype19 was compared with several *Plasmodium* species, PvKtype19 showed 89.5% identity with *P. falciparum* FCC1_HN (DQ825436), 90.2% with *P. falciparum* Mzr-1 (JN54719), 90.2% with *P. falciparum* Ori-1 (JN547218), 90.2% with *P. berghei* (AY437808), 96.8% with *Plasmodium knowlesi* (JF958130), and 90.2% with *Plasmodium reichenowi* (AB122147) (Figures 4 and 5). When the amino acid sequence of PvKtype19 was compared with several *P. vivax* strains, PvKtype19 showed 100% identity with *P. vivax* Hainan (FJ527750), 97.8% with *P. vivax* Ori-1 (JN547221), 99.7% with *P. vivax* Krt-1 (JN547225), and 98.4% with *P. vivax* Goa-1 (JN547223) (Figures 6 and 7).

### **Expression of pLDH in***Escherichia****coli***

**Figure 8:**
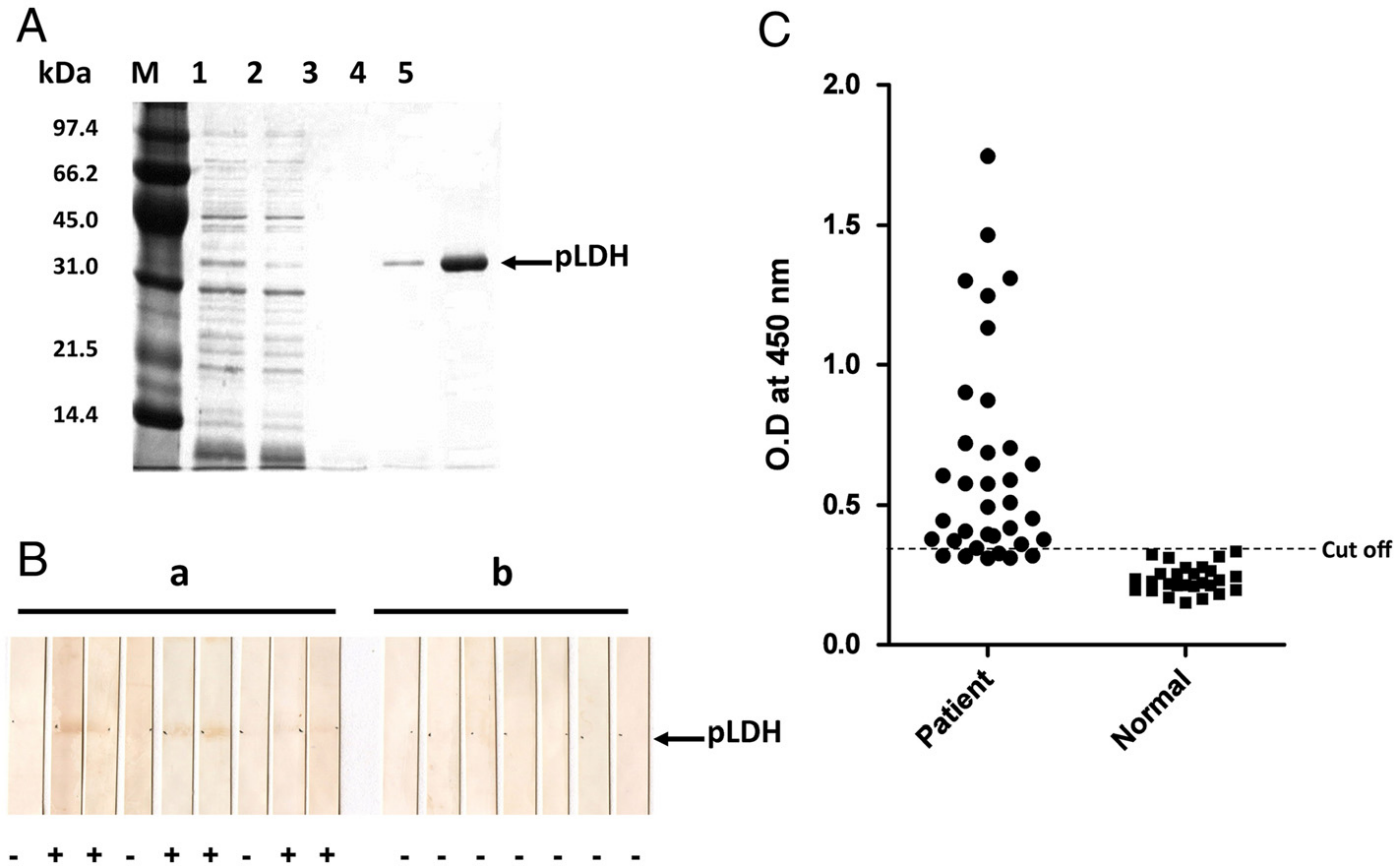
**A) Purification of pLDH with Ni-NTA agarose affinity chromatography.** Lane M, molecular weight protein marker; lane 1, induced *E. coli* DH5α cell lysate with IPTG; lane 2, flow-through; lane 3, wash; lane 4–5, elutes. **B**) Western blot analysis of pLDH recombinant protein. a, Malaria patients; b, Healthy individuals. **C**) Scattergram of absorbances measured by ELISA using pLDH recombinant protein. Sera from healthy individuals and malaria patients infected with *P. vivax* were used. Cut off showed the mean + 2 X SD of negative controls.

The resulting plasmid pVKtype19 contained a pLDH gene fused to a (His) _6_-tag based on pQE80 (Figure 1B). The recombinant plasmid pVKtype19 was then transferred into *E. coli* DH5α. As analysed by SDS-PAGE followed by Coomassie blue staining, the pLDH recombinant protein was 32 kDa under native purification conditions (Figure 8A).

### Antigenicity of the pLDH recombinant protein

To determine the antigenicity of the pLDH recombinant protein by western blot and ELISA, the sera of malaria patients that had been collected between 2009 and 2010, which confirmed by microscopic examination but did not count the parasites (parasitaemia), and kept by the KNIH were analysed. Negative sera were collected from staff volunteers from the KNIH.

The sera of six of nine malaria patients exhibited a positive reaction by western blot, while the sera from the normal control group (n = 7), who had never been exposed to malaria, tested negative (Figure 8B). After the number of malaria patients was increased, the antigenicity of the recombinant pLDH protein was evaluated by ELISA. Thirty-four of the 40 sera (85.0%) from malaria patients, as confirmed by microscopic analysis, reacted with the pLDH recombinant protein. In addition, all of the 26 samples from the normal control group failed to react with the pLDH recombinant protein (Figure 8C).

## Discussion

pLDH is one of the target antigens that is widely used in developing the monoclonal antibodies that are part of the RDT that comprises the non-microscopic immunochromatographic assay. Interestingly, the level of pLDH has been shown to decline in parallel with the clearance of asexual parasitaemia; therefore, it has been suggested that the disappearance of the parasite-specific enzyme pLDH after anti-malarial drugs may be useful in predicting treatment failure [23]. These characteristics of pLDH led us to investigate the sequence variability of pLDH in Korean isolates. PvKtype19, which was the predominant form of pLDH in Korean isolates, exhibited higher identity with *P. knowlesi* (96.8%, JF958130) than with *P. falciparum* Ori-1 (JN547218) (Figures 4 and 5). However, PvKtype19 showed 97.8-100% identity with other subspecies of *P. vivax* (Figures 6 and 7). Only one synonymous SNP was found in 20 Korean isolates, at base pair 456 (n = 1) (Figures 2 and 3).

*Plasmodium vivax* has presumably been prevalent in Korea for a long time. However, as a result of a national malaria eradication programme and with help from the World Health Organization (WHO), the incidence of vivax malaria has rapidly decreased [24,25]. After the latest report of two malaria patients in 1985 [26], there were no additional reported cases until one case was reported in 1993 [27] and two indigenous cases were reported in 1994 [28]. Malaria cases then rapidly increased until approximately 2000 [29,30]. After that, the reported malaria cases decreased for several years due to efforts to limit the incidence of malaria. However, malaria has not been thoroughly eradicated in the Korean peninsula because 2 to 3% of patients experience failed drug treatment every year, and many travellers and workers come from malaria-prevalent areas, including North Korea [31]. For these reasons, serological diagnostic tools are needed to support both traditional microscopic diagnosis and antibody testing on a population level, to get a proxy about exposure to malaria in Korea. Currently, IFAT (Immunofluorescence antibody test) is used as the standard serological diagnostic method due to its high sensitivity in this context. However, the sensitivity might be affected by the training and ability of examiners. Therefore, a new antigen is needed for serodiagnosis. Several recombinant proteins cloned from Korean isolates of *P. vivax* have been tested for use as antigens for serodiagnosis, including circumsporozoite protein (CSP), subtypes Pv210 [32] and Pv247 [33], merozoite surface protein (MSP) [34], and CSP and MSP chimeric proteins [35,36]. None of these antigens were capable of replacing the IFAT method because their sensitivity was less than that of IFAT. Therefore, it was decided to focus on pLDH. Monoclonal antibodies against pLDH have been used in several RDTs and exhibit a relatively high sensitivity for the detection of malaria parasites. However, the ELISA detected only 85.0% (34/40) of microscopic positive samples, even though it was cloned from a Korean vivax malaria strain (pVKtype19, Figure 5). Therefore, antibody detection using the pLDH recombinant protein is not sufficient to compensate the disadvantage of antigen detection using its monoclonal antibody. However, it should be investigated whether pLDH recombinant protein can detect asymptomatic patients or symptomatic patients who have low parasitaemia (under 50/μl) using by antibody detection methods, for example, ELISA or Western blot. Therefore, when using the RDT in the field, it is likely better to use both antigen and antibody detection RDTs to compensate for their individual limitation.

## Conclusions

The pLDH gene from *P. vivax* Korean isolates has an SNP at position 456 (T to C). New information from the geographic mapping of pLDH at the national or regional scale would provide a valuable aid for developing and updating the national anti-malarial policy guidelines in Korea. Additionally, more information is needed before using pLDH as a serological diagnostic antigen.

## Competing interests

The authors declare that they have no competing interests.

## Authors’ contributions

YJK, YS, HIS and HWL conceived and designed the study and contributed to the execution of the research. HWL wrote the manuscript. JYK, HIS and WJL collected the blood samples in the field. JYK and HIS performed the preparation of DNA samples for DNA sequencing. SWL, who had been working at the University of Florida as a volunteer, (Eastside High School) expressed recombinant pLDH and performed the western blot and ELISA. All authors have read and approved the final manuscript.

## References

[B1] Breman JG The ears of the hippopotamus: manifestations, determinants and estimates of the malaria burden Am J Trop Med Hyg 2001 64 1 11 11425172 10.4269/ajtmh.2001.64.1

[B2] Phillips RS Current status of malaria and potential for control Clinic Microbiol Rev 2001 14 208 226 10.1128/CMR.14.1.208-226.2001 PMC88970 11148010

[B3] Reyburn H Mbatia R Drakeley C Carneiro I Mwakasungula E Mwerinde O Saganda K Shao J Kitua A Olomi R Greenwood BM Whitty CJ Overdiagnosis of malaria in patients with severe febrile illness in Tanzania: a prospective study BMJ 2004 329 1212 10.1136/bmj.38251.658229.55 15542534 PMC529364

[B4] Iqbal J Siddique A Jameel M Hira PR Persistent histidine-rich protein 2, parasite lactate dehydrogenase, and panmalarial antigen reactivity after clearance of *Plasmodium falciparum* monoinfection J Clin Microbiol 2004 42 4237 4241 10.1128/JCM.42.9.4237-4241.2004 15365017 PMC516301

[B5] Wongsrichanalai C Rapid diagnostic techniques for malaria control Trends Parasitol 2001 17 307 309 10.1016/S1471-4922(01)01925-0 11423356

[B6] Moody A Rapid diagnostic tests for malaria parasites Clin Microbiol Rev 2002 15 66 78 10.1128/CMR.15.1.66-78.2002 11781267 PMC118060

[B7] Bell D Peeling RW Evaluation of rapid diagnostic tests: malaria Nat Rev Microbiol 2006 4 S34 S38 10.1038/nrmicro1524 17034070

[B8] Singh N Saxena A Valecha N Field evaluation of the ICT malaria P.f/P.v immunochromatographic test for diagnosis of *Plasmodium falciparum* and *P. vivax* infection in forest villages of Chhindwara, central India Trop Med Int Health 2000 5 765 770 10.1046/j.1365-3156.2000.00645.x 11123823

[B9] Cho CH Nam MH Kim JS Han ET Lee WJ Oh JS An SS Lim CS Genetic variability in *Plasmodium vivax* aldolase gene in Korean isolates and the sensitivity of the Binax Now malaria test Trop Med Int Health 2011 16 223 226 10.1111/j.1365-3156.2010.02691.x 21087378

[B10] Baker J McCarthy J Gatton M Kyle DE Belizario V Luchavez J Bell D Cheng Q Genetic diversity of *Plasmodium falciparum* histidine-rich protein 2 (PfHRP2) and its effect on the performance of PfHRP2-based rapid diagnostic tests J Infect Dis 2005 192 870 877 10.1086/432010 16088837

[B11] Brown WM Yowell CA Hoard A Vader Jagt TA Hunsaker LA Deck LM Royer RE Piper RC Dame JB Makler MT Vanderjagt DL Comparative structural analysis and kinetic properties of lactate dehydrogenases from the four species of human malarial parasites Biochem 2004 43 6219 6229 10.1021/bi049892w 15147206

[B12] Piper R Lebras J Wentworth L Hunt-Cooke A Houze S Chiodini P Marker M Immunocapture diagnostic assays for malaria using *Plasmodium* lactate dehydrogenase (pLDH) Am J Trop Med Hyg 1999 60 109 114 9988333 10.4269/ajtmh.1999.60.109

[B13] Sherman IW Molecular heterogeneity of lactic dehydrogenase in avian malaria (*Plasmodium lophurae*) J Exp Med 1961 114 1049 1062 10.1084/jem.114.6.1049 13911722 PMC2180406

[B14] Sherman IW Heterogeneity of lactic dehydrogenase in intraerythrocytic parasites Trans NY Acad Sci Ser 1962 2 24 944 953 10.1111/j.2164-0947.1962.tb01454.x 13977174

[B15] Rose EF Jr Raventos S Perkins M Nagel RL Glutathione stability and oxidative stress in *P. falciparum* infection in vitro: response of normal and G6PD deficient cells Biochem Biophys Res Commun 1982 109 355 10.1016/0006-291X(82)91728-4 6758788

[B16] Sherman IW Peters W, Richards WHG Metabolism Antimalarial drugs I. Biological background, experimental methods and drug resistance 1984 Heidelberg: Springer Verlag 31 81

[B17] Fairlamb AH Novel biochemical pathways in parasitic protozoa Parasitol 1989 99 S93 S112 10.1017/S003118200008344X 2682488

[B18] Farcas GA Zhong KJ Lovegrove FE Graham CM Kain KC Evaluation of the Binax Now ICT test versus polymerase chain reaction and microscopy for the detection of malaria in returning travellers Am J Trop Med Hyg 2003 69 589 592 14740873

[B19] World Health Organization A rapid dipstick antigen capture assay for the diagnosis of falciparum malaria: WHO informal consultation on recent advances in diagnostic techniques and vaccines for malaria Bull World Health Organ 1996 74 47 54 8653815 PMC2486846

[B20] Lee HW Lee WJ Lee JS Lee HS DNA sequencing and expression of the circumsporozoite protein of *Plasmodium vivax* Korean isolate in *Escherichia coli* Kor J Microbiol 1999 37 234 242

[B21] Tsang VCW Peralta JM Simons AR Enzyme-linked immunoelectrotransfer blot techniques (EITB) for studying the specificities of antigens and antibodies separated by gel electrophoresis Meth Enzymol 1983 92 377 391 6855619 10.1016/0076-6879(83)92032-3

[B22] Gao YH Li HL Lu Y Gao FM Lin YH Zhou HC Zhang LH Wang H Identification of a vaccine candidate antigen, PfMAg-1, from *Plasmodium falciparum* with monoclonal antibody M26-32 Parasitol Res 2009 105 1723 1732 10.1007/s00436-009-1617-4 19777263

[B23] Nyunt MH Kyaw MP Win KK Myint KM Nyunt KM Field evaluation of HRP2 and pan pLDH-based immunochromatographic assay in therapeutic monitoring of uncomplicated falciparum malaria in Myanmar Malar J 2013 12 123 10.1186/1475-2875-12-123 23577630 PMC3636062

[B24] National Malaria Eradication Service *Malaria pre-eradication program in Korea. Progress Report 1961–1965.* Ministry of Health and Social Affairs Republic of Korea 1966 44 70

[B25] Paik YH Ree HI Shim JC Malaria in Korea Jap J Exp Med 1988 58 55 66 3045377

[B26] Soh JT Lee KT Im KI Min DY Ahn MH Kim JJ Yong TS Current status of malaria in Korea Yonsei Rep Trop Med 1985 16 11 18

[B27] Chai IH Lim GI Yoon SN Oh WI Kim SJ Chai JY Occurrence of tertian malaria in a male patient who has never been abroad Kor J Parasitol 1994 32 195 200 10.3347/kjp.1994.32.3.195 7953245

[B28] Cho SY Kong Y Park SM Lee JS Lim YA Chae SL Kho WG Lee JS Shim JC Shin HK Two vivax malaria cases detected in Korea Kor J Parasitol 1994 32 281 284 10.3347/kjp.1994.32.4.281 7834248

[B29] Lee JS Kho WG Lee HW Seo M Lee WJ Current status of vivax malaria among civilians in Korea Kor J Parasitol 1998 36 241 248 10.3347/kjp.1998.36.4.241 PMC2732963 9868889

[B30] Park JW Klein TA Lee HC Pacha LA Ryu SH Yeom JS Moon SH Kim TS Chai JY Oh MD Choe KW Vivax malaria: a continuing health threat to the Republic of Korea Am J Trop Med Hyg 2003 69 159 167 13677372

[B31] Lee WJ Kim HH Choi YK Choi KM Kim MA Kim JY Sattabongkot J Sohn Y Kim H Lee JK Park HS Lee HW Analysis of the dihydrofolate reductase-thymidylate synthase gene sequences in *Plasmodium vivax* field isolates that failed chloroquine treatment Malar J 2010 9 331 10.1186/1475-2875-9-331 21087471 PMC2999615

[B32] Lee HW Lee JS Lee WJ Cho SH Lee HS The evaluation of recombinant circumsporozoite protein in malaria diagnosis Kor J Microbiol 2000 36 142 149

[B33] Kim TS Kim HH Lee SS Oh CM Choi KM Lin K Kim JY Na BK Han ET Sohn Y Kim H Lee HW Molecular cloning and expression of the VK247 circumsporozoite protein for serodiagnosis of variant form *Plasmodium vivax* Parasitol Res 2011 108 1275 1282 10.1007/s00436-010-2177-3 21318386

[B34] Kwon MH Kim HH Lee HS Kim TS Oh CM Ahn YJ Hwang SK Sohn Y Kim H Lee HW *Plasmodium vivax*: comparison of the immune responses between oral and parenteral immunization of rPv54 in BALB/c mice Exp Parasitol 2010 126 217 223 10.1016/j.exppara.2010.05.001 20460123

[B35] Lee C Chung KW Kim TS Choi KM Choi YK Chung NJ Rhie HG Lee HS Lee SJ Lee HW Trials for the co-expression of the merozoite surface protein-1 and circumsporozoite protein genes of *Plasmodium vivax* Exp Parasitol 2011 129 227 233 10.1016/j.exppara.2011.08.014 21907198

[B36] Lee C Kim HH Choi KM Chung KW Choi YK Jang MJ Kim TS Chung NJ Rhie HG Lee HS Sohn Y Kim H Lee SJ Lee HW Murine immune responses to a *Plasmodium vivax*-derived chimeric recombinant protein expressed in *Brassica napus* Malar J 2011 29 106 21529346 10.1186/1475-2875-10-106 PMC3098821

